# Pulmonary hypoplasia with vertebral anomaly

**DOI:** 10.11604/pamj.2024.48.66.43699

**Published:** 2024-06-25

**Authors:** Ashwin Karnan, Vivek Alone

**Affiliations:** 1 Department of Respiratory Medicine, Jawaharlal Nehru Medical College, Datta Meghe Institute of Higher Education and Research, Sawangi (Meghe), Wardha, Maharashtra, India

**Keywords:** Dyspnea, scoliosis, congenital anomaly

## Image in medicine

A 12-year-old girl born to a non-consanguineous marriage presented to the outpatient department with complaints of breathlessness, neck pain, and a history of recurrent respiratory tract infections. Computed tomography (CT) scan of the thorax showed hypoplasia of the right lung with mediastinal shift towards the right side with platybasia, fusion of multiple cervical vertebrae, and presence of bilateral cervical rib. Pulmonary function tests showed a restrictive pattern of lung disease. The patient was diagnosed with pulmonary hypoplasia. The patient improved with intravenous antibiotics, bronchodilators, and chest physiotherapy. The patient has been counseled about her chronic lung disease, the need for prophylactic vaccinations, and spine surgery depending on the neurological function and pain status.

**Figure 1:**
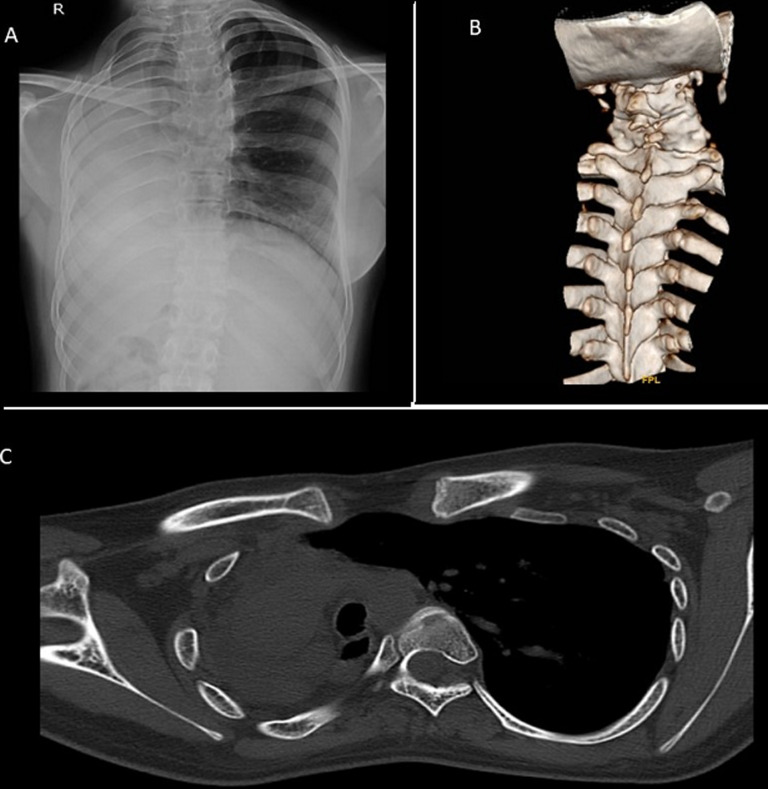
A) chest X-ray of the patient showing right opaque hemithorax with ipsilateral mediastinal shift; B) 3D reconstruction of the vertebra showing fusion of cervical vertebra suggesting segmental anomaly; C) computed tomography of thorax showing underdevelopment of the right lung with mediastinal shift to the right with compensatory hyperinflation and herniation of the left lung

